# Immunomodulation of Nanoparticles in Nanomedicine Applications

**DOI:** 10.1155/2014/426028

**Published:** 2014-05-20

**Authors:** Qing Jiao, Liwen Li, Qingxin Mu, Qiu Zhang

**Affiliations:** ^1^School of Chemistry and Chemical Engineering, Shandong University, Jinan 250100, China; ^2^Clinical Research Division, Fred Hutchinson Cancer Research Center, Seattle, WA 98109, USA; ^3^Department of Materials Science & Engineering, University of Washington, Seattle, WA 98125, USA

## Abstract

Nanoparticles (NPs) have promising applications in medicine. Immune system is an important protective system to defend organisms from non-self matters. NPs interact with the immune system and modulate its function, leading to immunosuppression or immunostimulation. These modulating effects may bring benefits or danger. Compositions, sizes, and surface chemistry, and so forth, affect these immunomodulations. Here we give an overview of the relationship between the physicochemical properties of NPs, which are candidates to be applied in medicine, and their immunomodulation properties.

## 1. Introduction

Large surface area, high aspect ratio, small size, and unique physical and chemical properties in NPs enable their potential applications in many biomedicine fields, such as drug and gene delivery, imaging, photodynamic therapy, and tissue engineering [1–3]. The small size of nanoparticls offers them the ability to overcome various biological barriers to transport and deliver therapeutic agents to the target tissue. NPs may overcome drug resistance when functionalized with targeting moiety [4–6]. The “nanophotosensitizers” used in photodynamic therapy (PDT) show higher solubility than normal photosensitizer playing an important role in the treatment of cancer [2]. Additionally, the increased resolution and sensitivity give nanostructure-based diagnostics an advantage over classical methods [7, 8]. Compared to traditional molecular medicine, NPs show advantages, such as intermixing, diffusion, sensoric response, and ultrafast kinetics make nanomedicine a local process at the nanoscale [9]. At the same time, NPs will enter and interact with human body during these processes.

As an important protective system to defend organisms from foreign matters and danger signals inside the body, the immune system plays a critical role in keeping homeostasis in human body. The immune system exerts its function through innate immunity and adaptive immunity. Innate immunity is the first line of defense against microbial invasion, which interacts with the foreign materials and cleans the pathogen or pathogen-infected cells, which is nonspecific to pathogen. The function of innate immunity was realized by the phagocytic cells (macrophages, dendritic cells (DCs), neutrophils, and mast cells (MCs), etc.), which phagocytose pathogen and release cytokine to clear pathogen. If the pathogen cannot be effectively cleared by innate immunity, the adaptive immunity, as the second line of defense in human body, will be activated. During these processes, some phagocytic cells act as antigen-presenting cells (APCs) and present specific antigens to specialized cells which are responsible for adaptive immunity, such as T cells and B cells. By this antigen-presenting process, pathogen (antigen) could be recognized by T cells and B cells and stimulate the adaptive immune response, which is specific to pathogen [10, 11]. The strong ability to eliminate pathogens makes the immune system important in most disease treatment. However, abnormal intensity of immune response, including immunosuppression and immunostimulation, will lead to disease [10]. Immunosuppression can be caused by impairment of any component of the immune system, which results in a decreased immune function and thereby leads to pathogen which cannot be effectively cleared and infection or tumor will occur [12]. Immunostimulation could enhance the ability to resist pathogen, but it may result in a strong adverse response such as autoimmune disease if it was hypersensitive.

**Figure 1:**
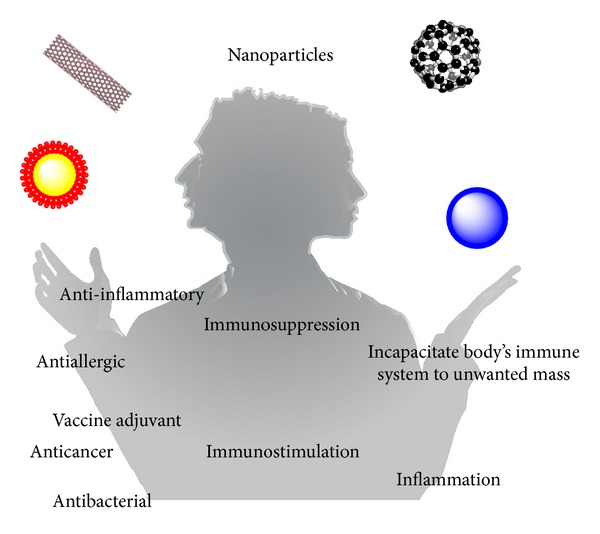
The immunomodulation of NPs presents a Janus' double-face in nanomedicine applications. On one hand, the effects to the immune system may benefit treatment of disease through enhancing immune response. On the other hand, the immunomodulation of NPs may bring harm.

When nanomedicines are applied* in vivo*, they act as foreign materials and induce the immune response, immunosuppression, or immunostimulation [13]. However, these modulations of immune system caused by NPs are undesirable in most cases when nanomedicine is applied, such as imaging. Furthermore, these immune modulations by NPs could be adverse in other conditions. Some nanobased anticancer therapeutic agents show antitumor properties* in vitro* but tumor-promoting effect* in vivo* [14]. This opposite effect may be due to the disturbed anticancer immune system [14]. However, some immunomodulation properties are good for disease prevention and treatment such as vaccine adjuvant and antiallergy therapeutic agents [15, 16]. Therefore, NPs play as a Janus' double-face in nanomedicine applications (Figure 1). Immunomodulating potential of NPs should be considered seriously because it could bring unexpected side effects in the clinical treatment. Understanding of nano-immuno-interactions is critical for the safe application of engineered NPs in medicine and safe design of nanomedicine.

In this review, we focus on the immunomodulating effects of NPs used in nanomedicine on immune system (Table 1). Effects of physicochemical properties of NPs on immune interactions and the underlying mechanisms are also reviewed.

## 2. NPs Candidates Used in Nanomedicine

Nanotechnology has a great potential in medicine applications such as medical diagnostics [60] and therapy [61]. As an inorganic fluorophore, quantum dots (QDs) have photostability which makes them ideal candidates for imaging tools* in vivo* [62]. Recent study showed a technique to track lymph flow in real time using quantum dots optical imaging in mice [22]. In addition, superparamagnetic iron oxide NPs (SPION) were also applied to trace neurodegenerative diseases by magnetic resonance imaging (MRI) [63]. Some carbon-based NPs are also applied in clinical use. Carbon nanotubes (CNTs) have unique physical properties such as electrical, thermal, and spectroscopic properties, which make them an advantage in detection and therapy of diseases [64]. It was reported that CNTs could prolong survival of tumor-bearing animals [65]. Graphene has good biocompatibility, biofunctionalization, and its unique mechanical, electronic, and optical properties for imaging and cancer phototherapy [66]. And it was demonstrated that graphene oxide (GO) have antibacterial properties [67], making them candidates as antibacterial agent. Besides, graphene derivatives are also good candidates for drug delivery as they can bind with aromatic drugs through *π*-*π* stack and/or van der Waals interactions [66]. Gold NPs (GNPs) are also potential materials in cancer therapies and imaging due to their biocompatibility, plasmon resonances, and diverse functionalizations [68]. It is promising to apply GNPs to targeted therapy of cancer [69] and overcome drug resistance [6]. Silver NPs (AgNPs) are important metal nanomaterial. They have antibacterial, antifungal, and antiviral effects [70]. Lipid NPs and liposome have been widely applied for drug delivery because of their improved drug potency and low off-target effects [71]. Other NPs such as polymer, CeO_2_, silica NPs, dendrimer, and protein NPs are also used in nanomedicine [72–78].

As foreign materials, NPs could be recognized by the immune system and induce immunosuppression or immunostimulation when used as nanomedicine. How to utilize or control these immunomodulation effects is largely based on NPs' different applications. NPs with immunosuppression effects might be used as anti-inflammatory or antiautoimmune disease therapeutic agents. On the contrast, NPs which activate immune system might be used as vaccines, or vaccine adjuvants. An advanced nanomedicine in drug delivery or imaging should not induce undesired immune-activation or immunosuppression effect. The detailed immunomodulation effects of these NPs in nanomedicine applications are discussed below.

## 3. Immunomodulation by Different NPs

### 3.1. Immunosuppression

#### 3.1.1. Carbon Nanotubes

After inhalation exposure, CNTs induced systemic immunosuppression in mice, including production of prostaglandin and IL-10 [17, 18] and T cell dysfunction [18, 19, 23]. For example, inhalation of CNTs (0.3, 1, or 5 mg/m³, 6 h/day, 14 days) hardly induced injury in lungs but resulted in nonmonotonic systemic immunosuppression (reduced T-cell-dependent antibody against sheep erythrocytes and T-cell proliferative ability and decreased natural killer cell activity). This suppression was accompanied by increased spleen gene expression of interleukin-10 (IL-10), which is an anti-inflammatory cytokine, and NAD(P)H oxidoreductase [17]. Other studies showed that pharyngeal aspiration of SWCNTs (40 *μ*g/mouse) in BALB/c mice induced pulmonary inflammation and suppressed the responsiveness of T cell 7 days postexposure. This immunosuppression was associated with the direct effects of SWCNTs on DCs [19].

#### 3.1.2. Fullerene

As a strong free-radical scavenger [79], fullerene has anti-inflammatory effects. The antioxidative/anti-inflammatory activities of novel fullerenes have been reported [80]. Fullerene could suppress Ag-driven type I hypersensitivity when human MCs and peripheral blood basophils were preincubated with C_60_ fullerenes. This suppression involved decreasing the level of reactive oxygen species (ROS) [16]. In a MC-dependent model of anaphylaxis, fullerenes prevented the release of histamine [16]. In addition, polyhydroxylated fullerene derivatives might protect against oxidative stress in ischemia-reperfused lungs [81]. C_60_ also suppressed the tumor necrosis factor alpha (TNF-*α*) induced production of proinflammatory cytokines* in vitro* and inhibited the arthritis* in vivo* [27]. Other studies in different animal models also showed the immunosuppression effects of fullerenes. For example, hydroxylated fullerenes could interfere with the innate immune system in fathead minnow [31]. Nanocrystalline fullerene showed cytotoxicity and promotive effects on tumor cell growth* in vitro* and* in vivo*, respectively, which might be due to the suppression of anticancer immune response of mice by fullerene [14].

#### 3.1.3. Gold NPs

The anti-inflammatory properties citrate-coated GNPs were also reported. Citrate coated GNPs (21 nm) did not cause detectable organ or cell toxicity in mice [37]. Studies also indicated that citrate-stabilized 5 nm and 15 nm GNPs inhibited cellular responses induced by interleukin 1 beta (IL-1*β*) and showed anti-inflammatory activity [38].

#### 3.1.4. Silver NPs

The studies on the immunotoxicity of AgNPs are very limited. AgNPs induced ROS and inflammation [82, 83], indicating its potential interference of immune system. AgNPs (22 nm) exposure caused the downregulation of expression of Malt1 and Sema7a genes, which were associated with immune cell function, followed by aberrant T cell differentiation [39].

#### 3.1.5. Magnetic NPs

Some* in vitro* studies showed that iron oxide NPs did not induce inflammatory response on human monocyte-macrophages [84] and aortic endothelia cells [85]. However, high doses of iron oxide NPs may induce oxidative stress [86]. When treated with PVA-coated SPION, human monocyte-derived DCs showed a decreased antigen processing and CD4 (+) T cell stimulation capacity [87]. These studies suggested the potential immune impact of magnetic NPs. The immunomodulation of the iron oxide NPs was much more complex* in vivo*. Intratracheally administration of high dose (4 × 500 *μ*g/mouse) and intermediate dose (4 × 250 *μ*g/mouse) of iron oxide NPs with a diameter of 35 ± 14 nm or 147 ± 48 nm inhibited the allergic Th2-dominated response induced by ovalbumin (OVA). The low dose (4 × 100 *μ*g/mouse) of iron oxide particles (147 ± 48 nm) had no significant effect, while the low dose (4 × 100 *μ*g/mouse) of particles (35 ± 14 nm) had an adjuvant effect on the Th2 response to OVA [45]. A single intratracheal instillation (250, 375 or 500 *μ*g/mouse) or four-time repeated instillation (500 *μ*g/mouse × 4) showed that both NPs induced lung inflammation and decreased pulmonary immune responses against sheep erythrocytes. In another study, intravenously administration of iron oxide NPs (58.7 nm) in doses ≤ 10 mg iron/kg shifted the Th1/Th2 immunobalance towards the Th2-dominant direction and suppressed the delayed-type hypersensitivity in OVA-sensitized mice [44]. Furthermore, repeated instillations resulted in a reduction of inflammation than single instillation [88].

#### 3.1.6. CeO_2_ NPs

Due to their reducibility, cerium oxide NPs (nanoceria) were found to have the ability to reduce ROS and may be used as a novel therapeutic tool for inflammation treatment [73]. Some* in vitro* studies indicated that nanoceria with a small diameter caused a significant anti-inflammatory effect [73, 89, 90]. For example, nanoceria with a diameter of 3–5 nm scavenged free radicals inhibited inflammatory mediator production in J774A.1, the murine macrophages [73]. A recent study reported that the same size of nanoceria induced APCs to secrete IL-10, and induced a Th2-dominated T cell proliferation. The nanoceria (5–8 nm) showed an effective antioxidant property in cardiac progenitor cells and protected the cardiac progenitor cells from H_2_O_2_-induced cytotoxicity. In addition,* in vivo* investigation on immune cells of the sea urchin indicated that nanoceria suppressed the innate immunity when force-fed 1 mL 10^−2^ g/L 50–60 nm of nanoceria [91].

#### 3.1.7. Quantum Dots

As efficient energy donors [92], QDs can induce the generation of ROS by transferring energy to nearby oxygen molecules.* In vitro* studies have shown that QDs induced production of ROS and led to multiple organelle damage and cell death [93, 94]. Preexposure to a dose at 10^−7^ to 10^−3^ *μ*g/mL CdTe QDs suppressed the immune responses of J774A.1 macrophage to bacteria by reducing NO, TNF-*α*, KC/CXCL-1, and IL-8 production [95]. These* in vitro* studies suggested that QDs might have high immunotoxicity.* In vivo* studies also showed similar results. CdTe QDs (1.6, 4, and 8 mg/L for 24 h at 15°C) significantly decreased the viability of hemocytes, as well as number of hemocytes capable of ingesting fluorescent beads in* Elliption complanata* mussels [50]. The immunosuppression was also observed in Juvenile rainbow trout. When 5, 10 and 20 nM CdS/CdTe QDs were exposed to Juvenile rainbow trout for 96 h at 15°C, the leukocyte counts, viability, and both resting and active phagocytic activity were significantly reduced [51]. QDs affected the proliferation of immune cells, but did not induce immune response including cytokine production [96]. Size of QDs aggregates may affect the immune response of QDs. Large CdS/CdTe QDs aggregates (25–100 nm) reduced phagocytosis more than smaller NPs (<25 nm) on bivalves (*Mytilus edulis* and* Elliptio complanata*) and fish (*Oncorhynchus mykiss*) [97]. Therefore, caution is needed to overcome this barrier when QDs are applied in clinical treatment.

#### 3.1.8. Polymeric NPs

*In vivo* studies indicated that some polymeric NPs inhibited inflammation but had no effect on host immunity [54, 98]. NPs produced by particle replication in nonwetting template technology remained in the lungs for up to 7 days without triggering host immunity after intratracheal administration of 50 *μ*g/mouse [98]. Polystyrene NPs (50 nm) inhibited lung inflammation by intratracheal administration of 200 *μ*g/mouse after allergen challenge. This inhibition was due to the modulation of DCs functions. NPs inhibited expansion of CD11c^+^MHCII^hi^ DCs in the lungs and draining lymph node and allergen-laden CD11b^hi^MHCII^hi^ DCs in the lungs [54]. In addition, polystyrene particles have the potential to halt the disease process in autoimmunity. For example, antigen-decorated polystyrene particles with a diameter of 500 nm induced T-cell tolerance and ameliorated experimental autoimmune encephalomyelitis by inactivating pathogenic T cells [55].

### 3.2. Immunostimulation

#### 3.2.1. Carbon Nanotubes

CNTs induced immunostimulation* in vitro* and* in vivo*. The oropharyngeal aspiration of MWCNTs (1, 2, and 4 mg/kg) in C57BL/6 mice induced inflammation (30 days postinstillation) in lungs [20, 21]. MWCNTs were translocated progressively into the spleen reached a maximum of 48 h after intraperitoneally (i.p.) administration, which caused the lymphocytic hyperplasia and increased oxidative stress in the spleen [99]. Subcutaneous administration of MWCNTs with total dose of 1.0 mg for two s.c injections in BALB/c mice induced acute immunological reactions for 1 week (activation of complement and increased proinflammatory cytokines). However, the accumulation of MWCNTs and injury was not observed in spleen [24]. MWCNTs injected intravenously activated Th2 immune response by elevating Th2 cytokines and increasing number of CD4^+^ and CD8^+^ T cell in the spleen [23]. In other studies, CNTs showed allergy adjuvant effect in inflammatory mass. For example, SWCNTs and MWCNTs exhibited adjuvant activity to the OVA-sensitized mice [25, 100]. MWCNTs aggravated asthma and induced fibrosis in OVA-sensitized lungs but showed no response with healthy pulmonary. The results indicated that these NPs could bring harm to asthma patients but not health ones [25]. In recent* in vitro* studies, MWCNTs increased the release of a series of cytokines in peripheral blood mononuclear cells (PBMCs) from healthy donors after stimulation with toll-like receptor (TLR) agonists or T cell mitogen. However, MWCNTs suppressed immune responses in PBMCs from mite-allergic subjects. These studies suggest that MWCNTs may either stimulate or suppress immune system depending on their immune cell target [101].

#### 3.2.2. Graphene

GO could induce healthy DCs to differention and maturation at varying degrees [102] but suppress the antigen-delivering ability of OVA-loaded DCs to T lymphocytes [103]. This inhibition was associated with downregulation of subunit LMP7 of immunoproteasome in cells, which is responsible for antigens processing in DCs [103]. When macrophage cells RAW264.7 were incubated with GO, toll-like receptor (TLR4/TLR9-) 6 modulated autophagy and inflammatory responses occurred [104]. In addition, PVP-coated GO exhibited lower immunogenicity than GO on the aspect of inducing maturation and differentiation of DCs [102]. PVP-coated GO enhanced the physiological activity of macrophages, which showed anti-phagocytosis ability against macrophages and delayed the apoptotic process of T lymphocytes [102]. This advantage makes PVP-coated GO a promising candidate of immunoadjuvant. The effect of two sizes (2 *μ*m and 350 nm) of the GO in response to microphages was investigated [105]. These two NPs had equal uptake amount in macrophages, but microsized GO induced stronger inflammation responses and showed divergent intracellular locations compared to nanosized GO [105]. This result demonstrated that lateral dimension of GO plays an important role in the regulation of cellular responses. Recent studies demonstrated that intravenously delivered graphene nanosheets induced site-specific Th2 inflammatory responses in the lungs via the IL-33/ST2 axis [23]. This effect may cause host defense and exacerbation of allergic diseases. However, more studies* in vivo* are needed to assess and eliminate the potential immunomodulation of graphene-based materials to ensure their safety for applications in biomedicine.

#### 3.2.3. Fullerene

Some studies showed that C_60_ have immunostimulatory properties [28–30, 103, 106]. After instillation, C_60_ upregulated gene expression of various proinflammatory cytokines (IL-1, TNF-*α*, IL-6) and Th1 cytokines (IL-12,IFN-*γ*) in mice. Besides inflammation, C_60_ could activate the immune system. The carboxyfullerene could prolong the infiltrating neutrophils to enhance the bactericidal activity of neutrophils [30]. Other studies indicated that fullerene may enhance the ability of DCs to stimulate T cells and furthermore activated cells of innate immune system by enhancing production of IL-6 and an activation of natural killer (NK) cells [103, 106]. In addition, immunization of mice with a C_60_ fullerene derivative conjugated to bovine thyroglobulin could produce a population of fullerene-specific antibodies, which included a subpopulation that cross-reacted with a C_70_ fullerene [29].

#### 3.2.4. Gold NPs

GNPs with different surface modification showed different immunogenicity in organisms. The immunogenicity of GNPs coated with C-terminal 19 kDa fragment of merozoite surface protein 1 (MSP-1_19_) was an important vaccine candidate. In this study, GNPs showed poor immunogenicity in mice but enhanced antibody response when formulated with alum [15]. However, GNPs coated with monosaccharide or disaccharides could initiate the immune response by activating the macrophages [107]. Some studies indicated that high concentrations of PEG coated on GNPs could induce antibody production and trigger immune responses. High doses of injected PEG-coated GNPs were cleared through these mechanisms [32, 33].

GNPs can also induce inflammatory responses* in vivo*. Well-dispersed PEG-coated GNPs (13 nm) can be recognized by host defense mechanism and induce acute inflammation and apoptosis in the liver [34]. If inflamed tissues are exposed, stronger immune responses may be induced [108]. When exposed to sensitized mice, 40 nm GNPs could lead to a threefold increase in airway hyperreactivity and increase the number of neutrophils and macrophages [36].

#### 3.2.5. Silver NPs

Intratracheal instillation of AgNPs with a diameter of 52.25 ± 23.64 nm could enhance the respiratory immune function through oxidative stress and induced inflammation in the respiratory. When alveolar macrophages were activated by AgNPs to cause phagocytosis, the alveolar macrophages generated ROS and free radicals which resulted in oxidative stress. The normal function of alveolar macrophages and epithelial cells was subsequently affected. This led to oversecretion of cytokines and oxides, which then caused the stimulation of the respiratory immune function [40]. These opposite results may be due to the different diameters of NPs. Covalent conjugation of Ag to solid core nanobeads with different diameters ranging from 0.02 to 2 *μ*m was found localized into DCs (DEC205^+^, CD40^+^, CD86^+^) in draining lymph nodes and induced high levels of IFN-*γ* production and high antibody titers in tumor-bearing mice [41].

#### 3.2.6. CeO_2_ NPs

Instillation of 100 *μ*g nanoceria with a diameter of 8 nm in C57BL/6 mice revealed that the NPs induced inflammation in pulmonary system by activating MCs [46]. Other studies using bigger sizes showed the same effect. Single intratracheal instillation of 20 nm nanoceria at 0.15–7 mg/kg caused a dose-dependent inflammation and lung injury [47]. The intratracheal instillation of 20–30 nm nanoceria (24.1 m^2^/g) with doses of 50 and 150 cm^2^/mouse induced both acute and chronic neutrophilic/mildly cytotoxic inflammation [48]. Inhalation of 55 nm nanoceria with an average aerosol concentration of 641 mg/m^3^ for 4 h induced cytotoxicity via oxidative stress and led to a chronic inflammatory response including up regulation of IL-1*β*, TNF-*α* and IL-6 [49].

#### 3.2.7. Silica NPs

30 nm amorphous silica NPs were investigated in PBMCs and purified monocytes. These NPs could induce inflammatory response as production of IL-1*β*, IL-8, and ROS. This result indicated the potential of silica NPs to evoke innate immune reactions [109]. Other reports showed that 30 and 70 nm silica NPs induced higher production of TNF-*α* in RAW264.7 cells and stronger inflammatory responses (IL-5↑, IL-6↑, MCP-1↑, keratinocyte chemoattractant↑) than 300 and 1000 nm particles* in vivo* through intraperitoneally injection. The 70 nm particle-induced TNF-*α* production was dependent on the production of ROS and activation of mitogen activated protein kinases (MAPKs). However, modification of carboxyl groups on 70 nm particles dramatically suppressed the inflammatory responses [52]. As well, Kupffer cells stimulated by 15 nm silica NPs released large amounts of ROS, TNF-*α* and NO. The viability of Buffalo rat liver (BRL) cells was reduced after cultured with silica NP-stimulated Kupffer cells. The authors also studied the intravenous injections of silica NPs with a single dose of 50 mg/kg. It caused hepatic inflammation and oxidative stress [53].

#### 3.2.8. Polymeric NPs

Polymer-based NPs were shown to be effective adjuvants in vaccination [72, 110, 111]. These polymers have the ability to activate cellular immune responses in the host [112]. For example, N-trimethyl chitosan-mono-N-carboxymethyl chitosan (TMC/MCC) NPs appeared to be very promising as an adjuvant and delivery system for antigens. Intranasal vaccination with tetanus toxoid loaded TMC/MCC NPs (283.5 ± 2.5 nm) was shown to induce both the mucosal and systemic immune response (enhanced antibody response). The enhanced immunoglobulin G (IgG) immune response could be explained by the sustained release of the toxoid [113].

Some polymer NPs were reported to activate immune system through modulating the activation and capability of immune cells, such as dendritic cells and T cells [114–116]. Amphiphilic NPs possessed pathogen-mimicking properties by activating DCs similar to lipopolysaccharide (LPS); thus, it has the ability to activate innate immune response [114]. Poly(methyl vinyl ether-co-maleic anhydride) NPs (149 ± 2 nm) activated DCs through TLR stimulation in innate immune system [115]. As well, the sulfonate (245 nm) and phosphonate-functionalized (227 nm) polystyrene NPs induced the maturation of immature DCs and significantly enhanced T cells stimulatory capacity, indicating a shift to Th1 response [116].

#### 3.2.9. Other NPs

Dendrimers have the ability to stimulate immune system and can be used as potential candidates for vaccines [76]. Maltose-functionalized dendrimer-peptide complex is a potential DC-based vaccine candidate by stimulating DC and activating the immune system [117]. Research also showed that Pan-DR-binding epitope-derivatized-dendrimer could reduce the effective dose of liposomal amphotericin B in murine cutaneous leishmaniasis and enhance adaptive immunity by activating strong parasite specific T-cell responses [56].

Protein NPs have shown immunostimulating properties in recent studies [118–120]. Self-assembled protein NPs that displayed epitopes of the repeat sequence in circumsporozoite protein of plasmodium falciparum (PfCSP) elicited a strong immune response against PfCSP [119, 121]. In addition, protein NPs mimic viruses have the ability to facilitate DCs activation and cross-presentation. These protein NPs codelivered with peptide epitopes to DCs showed an increased and prolonged CD8^+^ T cell activation [120].

A lipid NP was investigated in a DNA vaccine application. This NP which decorated with stearyl-conjugated KALA, an *α*-helical peptide (sequence WEAKLAKALAKALAKHLAKALAKALKACEA) showed enhancement of transgene expression; this enhancement was closely related to immune-activation [122]. A cationic solid-lipid NP was used as a vaccine to deliver a DNA vaccine against visceral leishmaniasis. High levels of IFN-*γ* and low levels of IL-10 production were detected in BALB/c mice after administration of the DNA vaccine delivered by this cationic solid-lipid NP. This NP induced a strong Th1 immune response, indicating its potential as therapeutic agent against visceral leishmaniasis [57]. Nanolipoprotein conjugated with TLR agonists monophosphoryl lipid A or CpG oligodeoxynucleotides significantly enhanced the immunostimulatory profiles compared to the agonists alone. Moreover, the BALB/c mice pretreated with CpG/nanolipoprotein coloaded nanoconstructs, but not CpG alone, survived a lethal influenza challenge [58]. Research also indicated that intravenous injection of liposome-DNA complexes elicited production of IFN-*α* and IFN-*β in vivo*, which suggested that the liposome-DNA complexes can induce inflammation and cause systemic toxicity [123]. In another study, it was reported that Doxil, an PEGylated liposomal formulation of doxorubicin, may cause hypersensitivity reactions while the standard doxorubicin did not, indicating that liposomes might be responsible for this hypersensitivity [59]. It was speculated that the complement system activation by Doxil may play a key role in this effect [59].

## 4. The Factors Affecting Immunomodulation of NPs

Many factors contribute to immunomodulation of NPs. The nature of NPs such as composition, surface chemistry, size, shape, and protein-binding ability dominates these interactions. Besides, individual difference and exposure route also contribute to immunomodulation of NPs.

### 4.1. Composition

Composition of NPs lays a vital role in the interactions between NPs and immune system. For example, QDs showed high immunotoxicity [50, 51] because they release heavy metal ions. Some other NPs exhibit less immunotoxicity [17, 23, 39, 44, 54], immunogenic [15, 32], or no effect [45]. The different core of NPs gave different reaction in allergy mass. For example, CNTs and GNPs showed adjuvant effect and led to hypersensitivity in these allergy masses [25, 26, 36] while fullerene often showed immunosuppression [14, 31].

### 4.2. Surface Chemistry

For NPs with the same composition, surface properties may also affect the immune system. Engineered NPs such as CNTs, fullerenes, GNPs, and silica NPs can be modified with diverse surface chemistry. This may alter their immune response both* in vitro* and* in vivo*.

**Figure 2:**
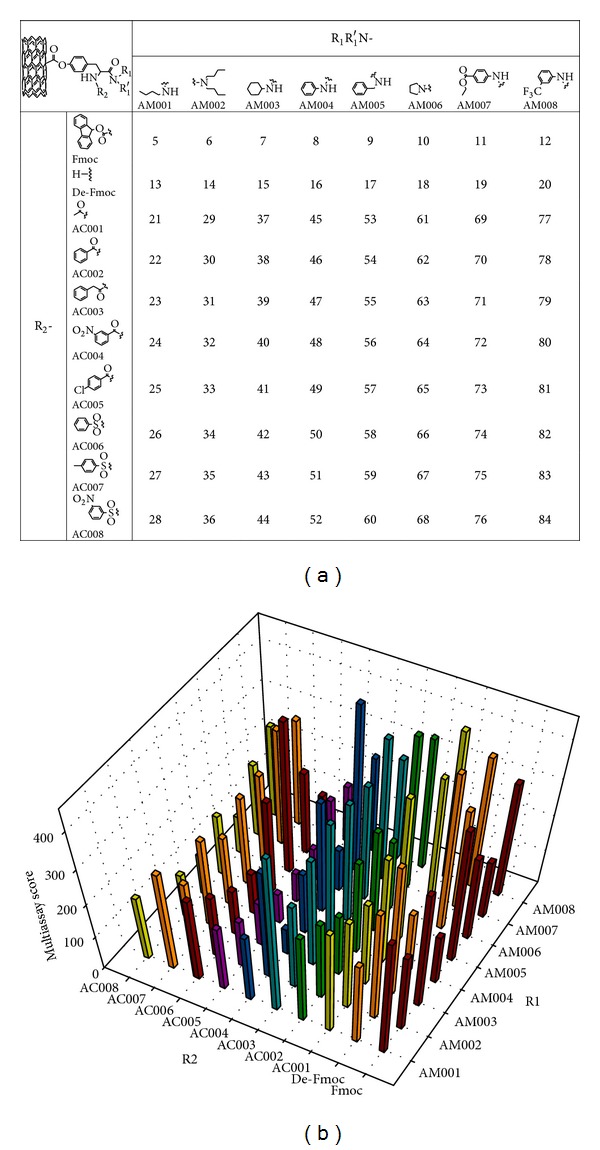
Multiassay score of the functional MWNT library. (a) Surface molecular compositions of combinatorial MWNT library members. (b) Findings from four protein (BSA, carbonic anhydrase, chymotrypsin, and hemoglobin) binding assays, cytotoxicity, and immune response assays (MWNT-induced NO release) at 50 *μ*g/mL in THP-1 macrophages were ranked for all library members. The sum of their ranks was designated the multiassay score and is shown as vertical bars in the graph. Reprinted with permission from [124].

Eighty diversely functionalized multiwall nanotube (MWNT) induced different levels of protein binding, cytotoxicity, and immune responses (Figure 2) [124]. The modification of MWCNTs significantly alleviated nuclear factor kappa B (NF-*κ*B) activation and reduced immunotoxicity of MWCNTs in BALB/c mice [125]. Carboxyfullerene activated immune system of C57BL/6 mice by prolonging the infiltrating neutrophils to enhance the bactericidal activity of neutrophils [30], while hydroxylated fullerenes interfered with the innate immune system in fathead minnow [31]. MWCNTs-PEG induced less generation of ROS and cytotoxicity in macrophages than MWCNTs-COOH, which was in correspondence with the lower cellular uptake of MWCNTs-PEG [126]. The monolayer-protected GNPs* in vivo* were studied. Simple place-exchange reactions within the monolayer by short chain, mercaptotetraethylene glycol, have been used. The short chain at lower concentrations did not trigger the immune system to produce anti-PEG antibody [33]. However, high concentration mixed monolayer coated NPs initiated an immune response [32]. Silica NPs (70 nm) induced strong inflammation by intraperitoneal injection, but these inflammatory responses could be dramatically suppressed by surface modification by carboxyl groups [52]. The toxicity of porous silica NPs to immune cells was surface chemistry and surface charge-dependent. Compare to surface hydrophobicity, surface charge had stronger impact on NPs' immunotoxicity* in vitro* and* in vivo*. Positively charged hydrophobic NPs showed more DNA damage than negatively charged hydrophilic NPs [127].

### 4.3. Size

Size is another important parameter that determines the interaction with organisms. When examined immunity is induced by a series of differentially sized (20, 40, 49, 67, 93, 101, and 123 nm) polystyrene nanobeads, IFN-*γ* induction from CD8^+^ T cells was limited to 40 and 49 nm beads, while 93–123 nm beads induced CD4^+^ T cell activation and increased IL-4 level. These results showed that the size of nanobead for vaccination could influence the type 1/type 2 cytokine balance. This would be useful in the development of vaccines against common human pathogens [128]. 200 nm NPs increased more antigen-specific polyfunctional CD4^+^ T cells as compared to 30 nm NPs. The immunoactivity of disaccharides coated GNPs is strongly dependent on size. They used 2 and 5 nm GNPs coated with disaccharides. These NPs activated the macrophages and induced the proliferation of T cells and the increase of IL-2 levels. The 5 nm NPs performed far better than 2 nm ones (increased APC proliferation, MHC II expression, T cell proliferation, and IL-2 expression) [107]. Other researches indicated that single instillation (250, 375, or 500 *μ*g/mouse) of 35 ± 14 nm iron oxide NPs induced higher levels of inflammation and immunodepression than 147 ± 48 nm ones [88]. Repeated intratracheal administration of high dose (4 × 500 *μ*g/mouse) and intermediate dose (4 × 250 *μ*g/mouse) of the same NPs inhibited the allergic Th2-dominated response induced by OVA. The low dose (4 × 100 *μ*g/mouse) of 147 ± 48 nm iron oxide particles had no significant effect, but the low dose (4 × 100 *μ*g/mouse) of 35 ± 14 nm particles had an adjuvant effect on the Th2 response to OVA [45]. Aggregate size may also affect the immunotoxicity of QDs. The toxicity of CdS/CdTe QDs was size dependent. Large CdS/CdTe QD aggregates (25 nm–100 nm) reduced phagocytosis of fish macrophages more than did smaller ones (<25 nm) [97].

### 4.4. Protein Binding

When NPs enter the body through injection, they firstly interact with blood [129]. The plasma protein binding on the NPs surface, such as apolipoprotein E and transferrin, may contribute to the activation/deactivation of receptor-dependent signaling [130].

The amount and types of proteins adsorbed on the NPs affect the interactions of cells and NPs and the biological responses [131, 132]. The composition, surface characteristics, and shape of NPs affect the manners that the proteins bind to them [129, 133–136]. The blood proteins adsorbed onto the NPs include immunoglobulins, apolipoproteins and proteins of the complement system among many others. These proteins may act as signals for immune responses [137, 138]. Furthermore, NPs may induce conformational changes in the structure of adsorbed proteins. Negatively charged poly(acrylic acid-) conjugated GNPs bound with fibrinogen and induced the unfolding of this protein, which promoted interaction with Mac-1, an integrin receptor. This activation increased the NF-*κ*B signalling pathway and released inflammatory cytokines [139].

### 4.5. Exposure Route

Exposure route is another factor affecting the immunomodulation of NPs. The outcomes of immune response are dependent on entrance of NPs. In the lungs, DCs, pulmonary epithelium, and macrophages play an important role in handling foreign materials. In the blood, leukocyte such as neutrophil, eosinophil, basophil, lymphocyte, and monocyte play a vital role. Single intratracheal instillation of 500 *μ*g/mouse iron oxide NPs (35 ± 14 nm and 147 ± 48 nm) induced lung inflammation and decreased pulmonary immune responses against sheep erythrocytes [88]. Pharyngeal aspiration of SWCNTs modified systemic immunity by modulating DCs function [19]. Intravenously administration of iron oxide NPs (58.7 nm) could suppress the infiltration and functional activity of Th1 cells and macrophages [44]. Intravenous injection of graphene nanosheets activated a Th2 immune response, which consisted of neutrophilic influx and a significant increase in IL-5, IL-13, IL-33 in the bronchoalvelar lavage fluid [23]. The dosage of administration is also important. Intratracheal injection of iron oxide NPs (35 ± 14 nm) inhibited the allergic response in OVA-sensitized mice at a dosage of 4 × 250 *μ*g/mouse or 4 × 500 *μ*g/mouse, but showed adjuvant effect at a dosage of 4 × 100 *μ*g/mouse [45].

## 5. The Mechanisms of Immunomodulation Induced by NPs

**Figure 3:**
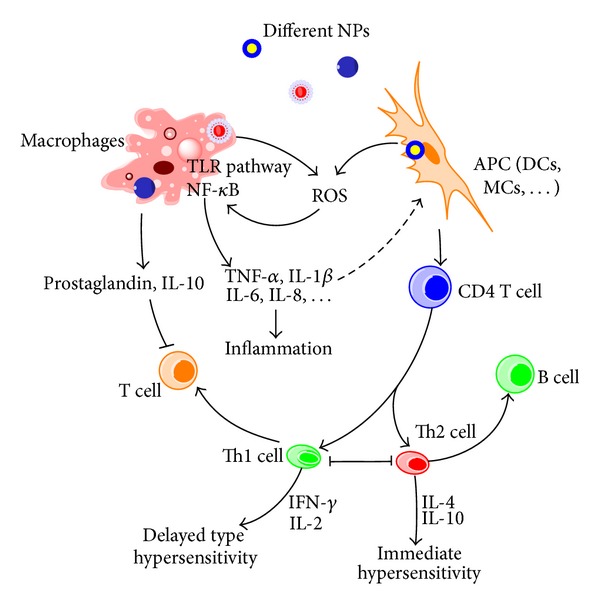
Mechanisms involved in NPs-induced immunomodulation. The stimulation/suppression to immune system depends on the nature of NPs and results in different outcomes. NPs, nanoparticles; NF-*κ*B, nuclear factor kappa B; TLR pathway: toll-like receptor pathway; APC, antigen-presenting cell; DCs, dendritic cells; MCs, mast cells; GM-CSF, granulocyte-macrophage colony-stimulating factor; Th0, type 0 T-helper lymphocyte; Th1, type 1 T-helper lymphocyte; Th2, type 2 T-helper lymphocyte; solid line with arrow, activate/release/induce; solid line with vertical dashes at ends, inhibit; dotted line, possible influence; broken line, polarization/differentiation.

NPs interact with both innate and adaptive immune cells, affect their functions, and disturb immune system (Figure 3).

Inflammation is an important response of immune system, which can be induced by NPs, evidenced by the production of cytokines or chemokines. Oxidative stress caused by NPs is reported to be the main downstream events of the inflammation. NPs have large surface areas and strong oxidative abilities than normal particles [140]. Oxidative damage induced by NPs is an important factor of immune imbalance [11]. Many types of NPs have been shown to produce ROS* in vitro* and* in vivo* and enhance immune function or inflammatory response [40, 49, 53, 86, 141]. Free radical-induced tissue damage plays an important role in inflammatory diseases [142, 143].

**Figure 4:**
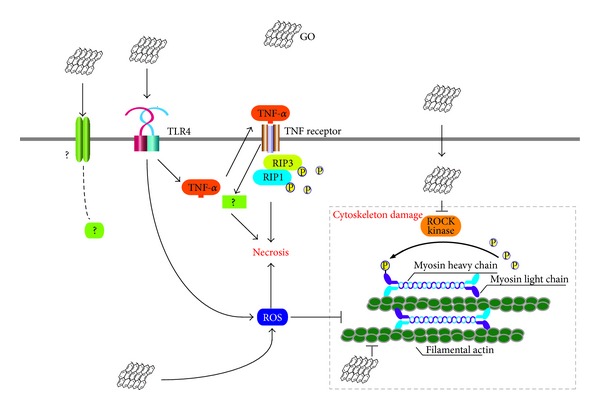
A schematic diagram elucidating the mechanisms responsible for GO-induced cytotoxicity to macrophages. Reprinted with permission from [144].

The signal pathway to induce ROS and mediate inflammation was reported. Among them, TLR4 signaling pathway was documented. GO could induce intracellular ROS which decreased the viability of macrophages and induced necrosis by a TLR4 signaling pathway (Figure 4) [144]. TLR is a receptor of the innate immune system and innate immunity could be triggered by stimulating TLRs and lead to strong adaptive immunity [115]. Activation of the TLR pathways could induce chronic inflammation and ROS [145]. Resent research indicated that quantum dots could activate myeloid differentiation primary response gene 88 (MyD88, which is an adapter protein to activate NF-*κ*B) dependent-TLRs in mcrophages and activated NF-*κ*B [146].

NF-*κ*B pathway is another key regulator of immune response [125, 147–149]. As an important regulator of proinflammatory gene expression, synthesis of cytokines such as IL-1*β*, IL-6, IL-8, and TNF-*α* is mediated by NF-*κ*B. Activation of NF-*κ*B may induce human inflammatory diseases [150, 151]. It was reported that negatively charged poly(acrylic acid-) conjugated GNP activated the NF-*κ*B signalling pathway in THP-1 cells. The cells released inflammatory cytokines including TNF-*α* and IL-8 [139]. And citrate-stabilized 10 nm GNPs could induce activation of an NF-*κ*B regulated luciferase reporter in murine B-lymphocyte cell line (CH12.LX) and altered the cell function [152].

NPs may also disturb the immune balance by inappropriate maturation/activation of APCs such as DCs [26, 153]. Intratracheal instillation of 43 nm MIONs, the alveolar region of BALB/c mice could generate a significant number of exosomes. These exosomes were quickly eliminated from the alveolar region into systemic circulation and transferred their signals to the immune system, which resulted in maturation of DCs and activation of splenic T cells, and the exosome-induced T-cell activation is more efficient in OVA-sensitized mice [42, 43]. Cytotoxicity to immune cells may contribute to immunosuppression of NPs. QDs may decrease the viability of hemocytes in* Elliption complanata* mussels [50] and reduced phagocytosis on bivalves and fish [97].

Recent reports indicated that NPs could modulate the homeostasis of immune cells, including the shift of Th1/Th2 balance [43, 44, 89] and monocyte homeostasis [154]. For example, magnetic iron oxide NPs activated the T cells, induced a Th1 polarization, and aggravated inflammation [43]. Other studies showed that SWCNTs accentuated Th cells immunity including Th2 (IL-4↑, IL-5↑, and IL-13↑) and Th17 (IL-17A↑, IL-23↑). The inappropriate maturation/activation of APCs such as DCs might be responsible for these accentuated Th cells immunity [26, 153]. The normal immune system keeps a Th1/Th2 balance in order to achieve an appropriate immune response. Selectively activating Th1 or Th2 cells results in immune deviation and breaks the balance of immune system. In addition, MWCNTs could selectively decrease phagocytosis-competent monocytes and promote adhesion of the phagocytosis-incompetent monocytes in blood flow [154].

## 6. Conclusion

The immune response of NPs is like a double-edged sword in nanomedicine applications by bringing both benefits and harms. We should take advantage of the benefits from the immunomodulating properties of NPs and, on the other hand, avoid the undesirable immune responses in order to minimize the systemic side effects. The factors affecting the immune response are complex, including particle composition, size, surface chemistry, plasma protein binding, and exposure route. Investigation of the relationship between properties of NPs and systemic immune response is crucial for their application in medicine and other areas. Although treatments of acute and long-term immune toxicities have been developed, current approaches of prediction, prevention, and treatment of nanoimmunomodulation are still lacking, encouraging further in-depth studies.

## Figures and Tables

**Table 1:** Immunomodulation of various nanoparticles in nanomedicine applications.

Nanomaterial	Size	Exposure routes/doses	Outcomes	Cytokines/chemokines	Animal	Reference
Carbon nanotube						
MWCNT	L: 5–15 *μ*m	Inhalation 5 mg/m^3^ 6 h/day 14 days	Immunosuppression	TGF*β*↑, IL-10↑	Male C57BL/6	[17, 18]
	D: 10–20 nm					
SWCNT	L: 1–3 *μ*m	Pharyngeal aspiration 40, 80, 120 *μ*g/mouse	Inflammation immunosuppression	TNF-*α*↑, IL-6↑, MCP1↑	Female BALB/c and C57BL/6	[19]
	D: 1–4 nm					
MWCNT	L: several *μ*m	Oropharyngeal aspiration 1, 2, and 4 mg/kg	Inflammation	IL-33↑, CCL3↑, CCL11↑	C57BL/6	[20, 21]
	D: 12.5–25 nm					
MWCNT	L: several *μ*m	Oropharyngeal aspiration 4 mg/kg	Inflammation	IL-33↑, IL-5↑, IL-8↑, IL-13↑	C57BL/6	[22]
	D: 12.5–25 nm					
MWCNT	L: 15 ± 5 *μ*m	Intravenously 1 mg/kg	Inflammation	IL-4↑, IL-33↑	C57BL/6	[23]
	D: 25 ± 5 nm					
MWCNT	L: 50 *μ*m	Subcutaneous 0.05, 0.3, and 0.5 mg × 2/mouse	Acute inflammation	IL-17↑, IL-1*β*↑, IL-1*α*↑, IFN-*γ*↑	BALB/c	[24]
	D: 20–30 nm					
MWCNT	L: 0.3–50 *μ*m	Inhalation 100 mg/m^3^ × 6 h	Hypersensitivity	PDGF-AA↑, TGF-*β*↑,	Allergic asthma mice (C57BL/6)	[25]
	D: 30–50 nm					
SWCNT	L: 3–30 *μ*m	Intratracheal 25, 50 *μ*g × 6/mouse	Hypersensitivity	IL-4↑, IL-5↑, IL-13↑, IFN-*γ*↑, IL-17A↑, IL-23↑, IL-33↑	Allergic inflammation mice (male ICR)	[26]
	D: 67 nm					

Graphene						
Graphene	4 ± 1 *μ*m^2^ area	Intravenously 1 mg/kg	Activate Th2 immune response	IL-33↑, IL-5↑, IL-13↑	C57BL/6	[23]
	2 ± 1 nm thick					

Fullerene						
C60	N/A	Intravenously 50 ng/mouse	Immunosuppression	Serum histamine↓, Lyn↓, Syk↓, ROS↓	MC-dependent model of anaphylaxis (C57BL/6)	[16]
C60	N/A	Intra-articular treatment 10.0 *μ*M/week × 8 week	Immunosuppression	TNF-*α*↓, IL-1*β*↓	Rat model of arthritis (female Sprague-Dawley rats)	[27]
C60	N/A	Instillation 2 mg/kg	Inflammation	IL-1↑, TNF-*α*↑, IL-6↑, IL-12↑, IFN-*γ*↑	Male ICR	[28, 29]
Carboxyfullerene	N/A	Peritoneum and air pouch 40 mg/kg	Activate immune system	N/A	C57BL/6	[30]
Hydroxylated C60	N/A	Intraperioneally injection 2 *μ*g/g	Immunosuppression	IL-11↑, elastase2 gene↓	Fathead minnow	[31]
C60	N/A	Intraperitoneal injection 0.5 mL × 10 *μ*g/mL × 14 days	Immunosuppression	IFN-*γ*↑	Tumor-bearing mice (C57BL/6)	[14]

Gold nanoparticles						
PfMSP-1_19_/PvMSP-1_19_ coated GNPs formulated with alum	17 nm	Subcutaneously 25 *μ*g/mouse	Immunogenic	Antibody titer↑	BALB/c	[15]
PfMSP-1_19_/PvMSP-1_19_ coated GNPs	17 nm	Subcutaneously 25 *μ*g/mouse	Poor immunogenic	N/A	BALB/c	[15]
Short-chain PEG mixed-monolayer protected gold clusters	<5 nm	Subcutaneously injection 40 *μ*M × 200 *μ*L	Immunogenic	Antibody titer↑	BALB/cAnNHsd	[32, 33]
PEG coated GNP	13 nm	Intravenously 0, 0.17, 0.85 or 4.26 mg/kg	Acute inflammation	MCP-1/CCL-2↑, MIP-1*α*/CCL-3↑, MIP-1*β*↑, RANTES/CCL-5↑, IL-1*β*↑, IL-6↑, IL-10↑, IL-12*β*↑, TNF-*α*↑	BALB/c	[34]
GNP functionalized with 2-mercaptoethanesulfonic acid (MES) or N,N,N-trimethylammoniumethanethiol (TMAT)	1.5 nm	Media exposure 0.016–250 ppm	Activate immune response Inflammatory response	Il-5↓, IL-12↓, IL-15↓, IL-18↓	Zebrafish embryos	[35]
Citrate-stabilized GNPs	40 nm	Oropharyngeal aspiration 0.8 mg/kg	Hypersensitivity	MMP-9↑, MIP-2↑, TNF-*α*↓, IL-6↓	TDI-sensitised mice (BALB/c)	[36]
GNP	21 nm	Intraperitoneally injection 7.85 *μ*g/g	Antiflammatory	TNF-*α*mRNA↓, IL-6 mRNA↓	Male C57BL/6	[37]
Citrate-stabilized GNPs	5 nm	100 nmol Au/kg	Antiflammatory	IL-1*β*↓	IL-1*β* model mice (male C57BL/6)	[38]

Silver Nanoparticles						
AgNP	22.18 ± 1.72 nm	Inhalation 1.91 × 10^7^ particles/cm^3^ × 6 h/day × 5 days/week × 2 weeks	Immunosuppression	Malt1 gene↓, Sema7a gene↓	C57BL/6	[39]
AgNP	52.25 ± 23.64 nm	Intratracheal instillation 3.5 or 17.5 mg/kg once every 2 days for 5 weeks	Enhance immune function	IL-1↑, IL-6↑, TNF-*α*↑, GSH↓, T-SOD↓, MDA↑, NO↑	Wistar rats	[40]
Ag conjugated to core nanobeads	40–50 nm	Intradermally	Immunogenic	IFN-*γ*↑, antibody↑	H-2K^b^ C57BL/6	[41]

Magnetic Nanoparticles						
Iron Oxide NP	43 nm	Intratracheal instillation (4 or 20 *μ*g × 3)	Activate immune response	IFN-*γ*↑, IL-4↑	OVA-sensitized mice (BALB/c)	[42, 43]
Iron Oxide NP	58.7 nm	Intravenously ≤10 mg iron/kg	Immunosuppression	IFN-*γ*↓, IL-6↓, TNF-*α*↓	DTH mice (male BALB/c)	[44]
Iron Oxide NP	35 ± 14 nm	Intratracheally 4 × 500 *μ*g/mouse Intratracheally 4 × 250 *μ*g/mouse	Immunosuppression	IgE↓, IL-4↓	OVA-sensitized mice (BALB/c)	[45]
		Intratracheally 4 × 100 *μ*g/mouse	Hypersensitivity	IgE↑, IL-4↑		
	147 ± 48 nm	Intratracheally 4 × 500 *μ*g/mouse Intratracheally 4 × 250 *μ*g/mouse	Immunosuppression	IgE↓, IL-4↓		
		Intratracheally 4 × 100 *μ*g/mouse	No significant effect	N/A		

Nanoceria						
Nanoceria	D: 8 nm	Oropharyngeal instillation of 10, 30, or 100 *μ*g/mouse	Inflammation	TNF-*α*↑, IL-6↑, osteopontin↑	C57BL/6	[46]
	A: 44 m^2^/g					
Nanoceria	20 nm	Single intratracheal instillation at 0.15–7 mg/kg	Inflammation	NO↓, IL-12↑	Specific pathogen-free male Sprague-Dawley (Hla: SD-CVF) rats	[47]
Nanoceria	D: 20–30 nm	Intratracheal instillation at 50 and 150 m^2^/mouse	Inflammation	IL-1*β*↑	Female Wistar rats	[48]
	A: 24.1 m^2^/g					
Nanoceria	D: 55 nm	Inhalation of 641 mg/m^3^ for 24 h, 48 h, and 14 days	Inflammation	IL-1*β*↑, TNF-*α*↑, IL-6↑, MDA↑, GSH↓	Wistar rats	[49]
	A: 30–50 m^2^/g					

Quantum Dots						
CdTe NP	N/A	1.6, 4, and 8 mg/L for 24 h	Immunosuppression	N/A	*Elliption complanata *	[50]
CdS/CdTe NP	N/A	5, 10 and 20 nm for 96 h	Immunosuppression	N/A	Juvenile rainbow trout	[51]

Silica Nanoparticles						
Amorphous silica NP	30 and 70 nm	Intraperitoneal injection of 1 mg/mice	Inflammation	IL-5↑, IL-6↑, MCP-1↑, keratinocyte chemoattractant↑	Female BALB/c	[52]
Amorphous silica NP modificated with carboxyl groups	70 nm	Intraperitoneal injection of 1 mg/mice	Suppression of inflammation	N/A		
Nonporous nanosilica NP	15 nm	Intravenous injections at single dose at 50 mg/kg	Inflammation oxidative stress	ROS↑, TNF-*α*↑, NO↑	Male SD rats	[53]

Polymer						
Polystyrene NP	50 nm	Intratracheal administration of 200 *μ*g/mouse	Anti-inflammation immunosuppression	IL-4↓, IL-5↓, IL-13↓	Allergen challenge mice	[54]
Polystyrene beads (PSB) coupled with the immunodominant myelin proteolipid protein PLP_139–151_ epitope (PLP_139–151_-PSB)	500 nm	intravenous injection of approximately 9 × 10^9^ microparticles	T-cell tolerance	IL-17↓, INF-*γ*↓	Peptide-induced experimental autoimmune encephalomyelitis SJL/J mice	[55]

Dendrimer						
Pan-DR-binding epitope (PADRE)-derivatized-dendrimer (PDD)	N/A	Intravenous injection with 6.25 mg/kg/day of LAmB at a ratio of 10 : 1 (PDD : LAmB) for 10 days	enhanced adaptive immunity	IFN-*γ*↑	Female BALB/c mice inoculated intraperitoneally with metacyclic promastigotes of L. major	[56]

Lipid Nanoparticles						
cSLN-pDNA (a DNA vaccine harbouring the *L*. *donovani A2 *antigen along with *L.infantum *cysteine proteinases) complexes	241 ± 12 nm	immunized in the right-hind footpad with 50 *μ*g of Qiagen purified pDNA	Enhanced immunity	Ratio of IFN-*γ* : IL-10↑	*L. infantum *promastigotes challenged female BALB/c mice	[57]
MPLA : NLP	6–25 nm	intraperitoneal injection ion of 1, 5, 10, 20 *μ*g/mouse	Enhanced immunostimulatory	IL-6↑, TNF-*α*↑, MIP-1*α*↑	Female BALB/c mice	[58]
CpG : NLP constructs	6–25 nm	intraperitoneal injection ion of 10, 20, 40 or 80 *μ*g/mouse	Enhanced immunostimulatory	IL-6↑, TNF-*α*↑, MIP-1*α*↑	Female BALB/c mice	[58]
Pegylated liposomal doxorubicin (Doxil)	85–100 nm	Infuse in accordance with the administration guideline of Doxil	Hypersensitivity reactions occurred in 45% of patients	N/A	Patients with solid tumors (*n* = 29) treated with Doxil for the first time	[59]

## Abbreviations

APC: Antigen-presenting cell
CNT: Carbon nanotube
SWCNT: Single walled carbon nanotube
MWCNT: Multiwalled carbon nanotube
DC: Dendritic cell
OVA: Ovalbumin
BSA: Bull serum albumin
TGF-*β*: Transforming growth factor beta
PBMCs: Peripheral blood mononuclear cells
Th cells: Helper T cells
IL: Interleukin
GO: Grapheme oxide
TRL: Toll-like receptor
PVP: Polyvinyl pyrrolidone
MC: Mast cell
ROS: Reactive oxygen species
TNF-*α*: Tumor necrosis factor alpha
IFN-*γ*: Interferon gamma
PEG: Polyethylene glycol
GNP: Gold nanoparticle
AgNP: Silver nanoparticle
QDs: Quantum dots
PfCSP: Circumsporozoite protein of plasmodium falciparum
LPS: Lipopolysaccharide
MCP-1: Monocyte chemoattractant protein-1
MAPKs: Mitogen activated protein kinases
NF-*κ*B: Nuclear factor kappa B
MIONs: Magnetic iron oxide nanoparticles
DNA: Deoxyribonucleic acid
MHC II: Major histocompatibility complex class II.
